# A novel immune model predicts the prognosis of mantle cell lymphoma

**DOI:** 10.3389/fmed.2026.1751344

**Published:** 2026-01-29

**Authors:** Xiaoyu Hao, Yuqi Zhang, Wenxin Qi, Mingxia Zhu, Jing Wang, Ping Yang, Weilong Zhang, Hongmei Jing

**Affiliations:** Department of Hematology, Peking University Third Hospital, Beijing, China

**Keywords:** biomarker, immune function, machine learning, mantle cell lymphoma, prognosis

## Abstract

**Background:**

Mantle Cell Lymphoma (MCL) is a subtype of B-cell lymphoma characterized by varied clinical manifestations. The immune status is associated with MCL’s development and outcome. This study aims to evaluate the prognostic value of peripheral cytokines and blood lymphocyte subsets in MCL patients.

**Methods:**

This retrospective study analyzed patients’ clinical characteristics, treatment strategies, progression-free survival (PFS), and overall survival (OS). Immune cell levels and cytokines were evaluated via peripheral blood flow cytometry. Prognostic models incorporating immune characteristics were developed using XGBoost algorithms.

**Results:**

The study involved 78 MCL patients with a median follow-up period of 40 months. The median PFS and median OS were 32 and 48 months. Univariate analysis linked poor PFS to factors including elevated β2-MG, Ann Arbor stage III-IV, low albumin levels (<35 g/L), high SUVmax (≥11), reduced T cells (<70.42%), reduced CD4 + T cells (<34.63%), and increased NK cells (≥8.37%). Factors linked to poor OS included the pleomorphic and blastoid subtypes, albumin below 35 g/L, and high SUVmax (≥11). Multivariate analysis identified high SUVmax (≥11) as an independent predictor of poor PFS and OS in MCL patients. XGBoost regression and classification models were developed to determine feature importance, highlighting five key features: SUVmax, LDH, IL-2, TNF-*α*, and CD4 + T cells. A prognostic model using these immune features was created to predict patients’ PFS, dividing them into high-risk and low-risk categories. This model showed superior discriminatory power compared to the MIPI and MIPI-C models and had comparable calibration ability.

**Conclusion:**

This study developed an innovative immune prognostic model for evaluating the prognosis of MCL patients, integrating immune factors with existing clinical features to improve prognostic evaluation.

## 1 Introduction

Mantle cell lymphoma (MCL) is a unique subtype of B-cell non-Hodgkin lymphoma (NHL) characterized by the chromosomal translocation t (11; 14). Predominantly affecting the elderly, MCL exhibits considerable variability in clinical and biological characteristics, as well as prognosis (1–3). It is categorized into three subtypes: classic, blastoid, and pleomorphic (4). Developing an accurate prognostic model is crucial for optimizing the management of MCL patients. The Mantle Cell Lymphoma International Prognostic Index (MIPI) is a traditional model that evaluates prognosis based on four clinical factors: age, lactate dehydrogenase (LDH) level, performance status, and leukocyte count (3, 5). MIPI is widely used and effectively predicts outcomes for MCL patients. The MIPI-C score enhances this model by adding the proliferation index ki-67%, categorizing patients into four risk groups with 5-year overall survival rates of 85, 72, 43, and 17%. This refinement provides a more precise prognosis for MCL (6).

The immune system plays a vital role in defending against tumor development and progression, showing a significant association with prognosis and treatment response in lymphoma patients (7). Within the tumor immune microenvironment (TIME), immune cell subsets and cytokines are involved in lymphoma progression through regulation of immune escape and inflammatory responses (8). Recent advancements in lymphoma immunotherapy have shown promise, such as CAR-T therapy, immunomodulatory drugs, bispecific T cell engagers, and immune checkpoint inhibitors (9–12). However, traditional MIPI and MIPI-C scores do not incorporate immune-related parameters, complicating the understanding of why patients with similar clinical characteristics have different prognoses and treatment responses. In clinical practice, peripheral blood lymphocyte subsets are utilized as biomarkers for assessing the immune status of MCL, with studies highlighting their prognostic significance in other lymphoma subtypes. Research has also explored the prognostic value of CD4 + T cells and CD8 + T cells in lymphoma patients (13). In B-cell Non-Hodgkin’s lymphoma, CD3 + CD8 + T lymphocytes have been associated with prognosis (14). Incorporating immune cell components and cytokines into prognostic models can optimize lymphoma patient management and improve clinical outcomes (15). Peripheral blood lymphocyte subsets and serum cytokines are accessible, convenient, and reproducible biomarkers. Integrating these immune indicators into prognostic models may provide new approaches for refining the prognostic assessment of MCL.

Extreme Gradient Boosting (XGBoost) is a robust ensemble learning algorithm recognized for its ability to handle non-linear feature relationships, evaluate feature importance, and resist overfitting. It is particularly useful in constructing medical prognosis models. Unlike traditional statistical methods, XGBoost systematically evaluates multi-dimensional indicators with its built-in feature importance module, which in this study identified five core prognostic features in MCL. This study aimed to develop a prognosis model that incorporates immune parameters through a machine learning-based feature screening strategy to guide personalized treatment and management of MCL.

## 2 Materials and methods

### 2.1 Patients

The retrospective study analyzed data from mantle cell lymphoma (MCL) patients at Peking University Third Hospital between January 2010 and November 2024. It included patients newly diagnosed with MCL who had a confirmed pathological diagnosis. Exclusion criteria were: (1) a history of other primary malignant tumors; (2) comorbid autoimmune diseases; (3) severe infections, such as septic shock or severe pneumonia. Baseline data collected encompassed age, gender, pathological subtype, stage, Eastern Cooperative Oncology Group performance status (ECOG) score, PET-CT results, B symptoms, bone marrow aspiration pathology, lymph node aspiration pathology, MIPI score, MIPI-C score, initial treatment method, and initial treatment efficacy. Additionally, peripheral blood lymphocyte subsets and serum cytokines were measured by flow cytometry at diagnosis. The primary endpoint of the study was progression-free survival (PFS), defined as the time from lymphoma diagnosis to disease progression, recurrence, or death from any cause. The secondary endpoint was overall survival (OS), defined as the time from diagnosis to death from any cause or to the last follow-up date.

### 2.2 Flow cytometric quantification of immune cell subsets

At least 4 mL of peripheral blood should be collected from patients with MCL at the initial diagnosis without treatment. After thoroughly mixing the blood with the lymphocyte subset analysis reagent, incubate in the dark for 15 min. Subsequently, hemolysin was added to lysate the sample to remove red blood cells, thoroughly mixed, and incubated at room temperature in the dark for 15 min. Immune cell subsets were detected by BD Biosciences flow cytometry, and data analysis was completed by BD FACSDiva software. The cell subsets tested include: Total T cells (CD3+), cytotoxic T cells (CD3 + CD8+), helper T cells (CD3 + CD4+), total lymphocytes (CD45+), natural killer (NK) cells (CD16 + CD56 + CD3-), γδT cells, early activated T cells (CD3 + CD69+), mid-activated T cells (CD3 + CD25 + ) and late-activated T cells (CD3 + HLA-DR+).

### 2.3 Cytokine analysis

Venous blood was collected from treatment-naive patients with MCL at the initial diagnosis into K2EDTA anticoagulant tubes. Samples were centrifuged at 1,000–2,000 g for 10 min at 4 °C to separate serum. The following cytokines were quantified: interleukin-2 (IL-2), interleukin-4 (IL-4), interleukin-6 (IL-6), interleukin-8 (IL-8), interleukin-9 (IL-9), interleukin-10 (IL-10), interleukin-17A (IL-17A), interferon-*α* (IFN-α), granulocyte colony-stimulating factor (G-CSF), and tumor necrosis factor-α (TNF-α).

### 2.4 Construction of the prognostic model

Data preprocessing is performed using Python 3.8. The dataset is divided randomly into a training set and a test set, following a 7:3 ratio. The training set serves the dual purpose of feature selection and model training. In contrast, the test set is designated for the initial evaluation of performance. Multiple Imputation (MI) handles missing values. All features are standardized with StandardScaler. Separate XGBoost models are trained for regression and classification tasks. XGBoost’s built-in feature importance assessment calculates feature importance scores, identifying core clinical and immune factors vital for predicting MCL prognosis. After normalization, these scores are sorted in descending order to select key features for the development of MCL prognostic model. Regression coefficients from Cox regression analysis are used to assign corresponding scores. Patients are divided into low-risk and high-risk groups based on the median risk scores. Survival curves are plotted using the Kaplan–Meier method, and the Log-rank test compares survival differences between groups. We used the bootstrap method with 1,000 resamplings of the full cohort to compute time-dependent area under the curve (AUC) and assess the prognostic performance of the immune scoring model. AUC analyses were performed in Python using the cumulative_dynamic_auc function from the scikit-survival package. This function addresses censoring mainly through the inverse probability of censoring weighting (IPCW) approach.

### 2.5 Statistical analysis

This study employs Python 3.8 and GraphPad Prism 9 for statistical analysis and visualization. Continuous variables including clinical indicators and immune parameters were tested for normality with the Shapiro–Wilk test. Normally distributed data are reported as mean ± standard deviation; nonnormal data are reported as median (interquartile range). Categorical variables are presented as counts and percentages. For the purpose of group comparisons, continuous immune variables were dichotomized into two groups based on optimal cutoff values determined by maximizing the log-rank test statistic. Between-group comparisons of categorical variables were performed using the Chi-square test. Univariate survival analysis utilizes the Kaplan–Meier method, with the Log-rank test applied to assess survival differences among groups. Multivariate survival analysis is conducted using the Cox proportional hazards regression model. Statistical significance is denoted by a *p*-value less than 0.05.

## 3 Results

### 3.1 Cutoff values of peripheral blood lymphocyte subsets

Optimal cutoff values for lymphocyte subsets and serum cytokines were determined by maximizing the Log-rank test statistic. For total T cells, the cutoff was set at 70.42%, with 55.1% of patients exceeding this percentage. CD4 + T cells had a threshold of 34.63%, surpassed by 29.5% of patients, while CD8 + T cells had a cutoff of 43.97%, with 28.2% exceeding it. The cutoff for a decreased proportion of γδT cells was 5.76%, exceeded by 47.4% of patients. NK cells had a cutoff of 8.37%, with 20.5% of patients above this level. Regarding cytokines, the IL-2 cutoff was 1.89 pg./mL, with 57.7% of patients exceeding this value. TNF-*α* had a cutoff of 2.16 pg./mL, with 57.7% above this level, and G-CSF had a cutoff of 2.58 pg./mL, with 61.5% of patients exceeding it.

### 3.2 Clinical characteristics of patients

Table 1 presents the clinical characteristics of 78 patients with MCL. The median follow-up was 40 months, with median PFS and OS at 32 and 48 months, respectively. Patient ages ranged from 34 to 87 years, with a median age of 59. Notably, 18 patients (23.1%) were over 65. Eleven patients (14.1%) had an ECOG performance status above 1 score. B symptoms were observed in 24.4% of patients. A Ki-67 index greater than 30% was observed in 52 patients (66.7%). Elevated Lactate Dehydrogenase (LDH) and β2-microglobulin (β2-MG) levels were found in 37.2 and 47.4% of patients, respectively. Serum albumin (ALB) levels were below normal in 15 patients (19.2%). At diagnosis, 70 patients (89.7%) were in stage IV of the Ann Arbor stage.

**Table 1:** Characteristics of the patients at baseline.

Characteristic	N (%)
Gender	
Male	64 (82.1%)
Female	14 (17.9%)
Age	
Median (range)	59 (34, 87)
<65	60 (76.9%)
≥65	18 (23.1%)
B symptoms	
No	59 (75.6%)
Yes	19 (24.4%)
Albumin	
<35 g/L	15 (19.2%)
≥35 g/L	63 (80.8%)
Stage	
I	0
II	3 (3.8%)
III	5 (6.5%)
IV	70 (89.7%)
Ki-67 index	
<30%	26 (33.3%)
≥30%	52 (66.7%)
ECOG performance status score	
0–1	67 (85.9%)
≥2	11 (14.1%)
β2-MG	
Normal	39 (50.0%)
Elevated	37 (47.4%)
LDH	
<250 U/L	49 (62.8%)
≥250 U/L	29 (37.2%)
MIPI	
Low risk	33 (42.3%)
Intermediate risk	27 (34.6%)
High risk	18 (23.1%)
MIPI-C	
Low and low-intermediate risk	40 (51.3%)
Intermediate-high and high risk	38 (48.7%)

ECOG, Eastern Cooperative Oncology Group performance status; MIPI, Mantle Cell Lymphoma International Prognostic Index; β2MG, β2-Microglobulin; LDH, Lactate Dehydrogenase.

Patients were categorized using the classical clinical risk rating systems, MIPI and MIPI-C. According to the MIPI system, 33 patients (42.3%) were classified as low risk, 27 patients (34.6%) as intermediate risk, and 18 patients (23.1%) as high risk. In contrast, the MIPI-C system identified 40 patients (51.3%) as low and low-intermediate risk, while 48.7% were classified as intermediate-high and high risk.

The first-line treatments included BR (Bendamustine + Rituximab), R-CHOP (Rituximab + Cyclophosphamide + Doxorubicin + Vincristine + Prednisone), HyperCVAD (Cyclophosphamide + Vincristine + Doxorubicin + Dexamethasone/Methotrexate + Cytarabine), R-EPOCH (Rituximab + Etoposide + Prednisone + Vincristine + Cyclophosphamide + Doxorubicin), VR-CAP (Bortezomib + Rituximab + Cyclophosphamide + Doxorubicin + Prednisone), IR (Ibrutinib + Rituximab), and ZR (Zanubrutinib + Rituximab). Specifically, 11 patients received BR, 48 patients received R-CHOP, 10 patients received R-EPOCH, and 2 patients received HyperCVAD. The remaining 7 patients were treated with IR, VR-CAP, ZR regimens, or their regimens were unknown. In this cohort, 14 patients (17.9%) underwent rituximab maintenance therapy following initial treatment. Additionally, 38 patients (50.7%) achieved complete remission (CR) after first-line treatment.

### 3.3 Immunological characteristics of different MIPI-C risk stratifications

The MIPI-C is a well-established prognostic scoring system for MCL that incorporates five independent clinical factors: age, ECOG performance status, LDH level, white blood cell count, and Ki-67 percentage. Using MIPI-C, 78 MCL patients were divided into two groups: low risk and high risk. The low-risk group includes individuals from the low-risk and low-intermediate risk categories, while the high-risk group comprises those classified as intermediate-high risk and high risk. Table 2 illustrates the immune characteristics of each group. The MIPI-C low-risk group had a significantly higher proportion of patients with CD4 + T cell levels ≥34.63% (46.2% vs. 12.8%, *p* = 0.002) and a higher percentage of patients with IL-2 levels ≥1.89 pg./mL (71.8% vs. 43.6%, *p* = 0.021) compared to the high-risk group. These results indicate a potential association between the proportion of CD4 + T cell, IL-2 levels and patient prognosis. However, no significant differences were observed in the proportions of CD8 + T cells, γδT cells, NK cells, or immune markers such as TNF-*α* and G-CSF across the MIPI-C risk categories.

**Table 2:** Immune characteristics of different risk stratifications of MIPI-C.

Immune parameter	MIPI-C		*p*-value
	Low risk	High risk	
CD4 + T cell			0.002
<34.63%	21(53.8%)	34(87.2%)	
≥34.63%	18(46.2%)	5(12.8%)	
CD8 + T cell			0.451
<43.97%	30(76.9%)	26(66.7%)	
≥43.97%	9(23.1%)	13(33.3%)	
γδT cell			0.650
<5.76%	19(48.7%)	22(56.4%)	
≥5.76%	20(51.3%)	17(43.6%)	
NK cell			0.160
<8.37%	34(87.2%)	28(71.8%)	
≥8.37%	5(12.8%)	11(28.2%)	
TNF-α			0.066
<2.16 pg./mL	12(30.8%)	21(53.8%)	
≥2.16 pg./mL	27(69.2%)	18(46.2%)	
IL-2			0.021
<1.89 pg./mL	11(28.2%)	22(56.4%)	
≥1.89 pg./mL	28(71.8%)	17(43.6%)	
G-CSF			0.244
<2.58 pg./mL	12(30.8%)	18(46.2%)	
≥2.58 pg./mL	27(69.2%)	21(53.8%)	

NK cell, Natural Killer Cell; G-CSF, Granulocyte Colony-Stimulating Factor.

### 3.4 Prognostic factors of mantle cell lymphoma

**Figure 1:**
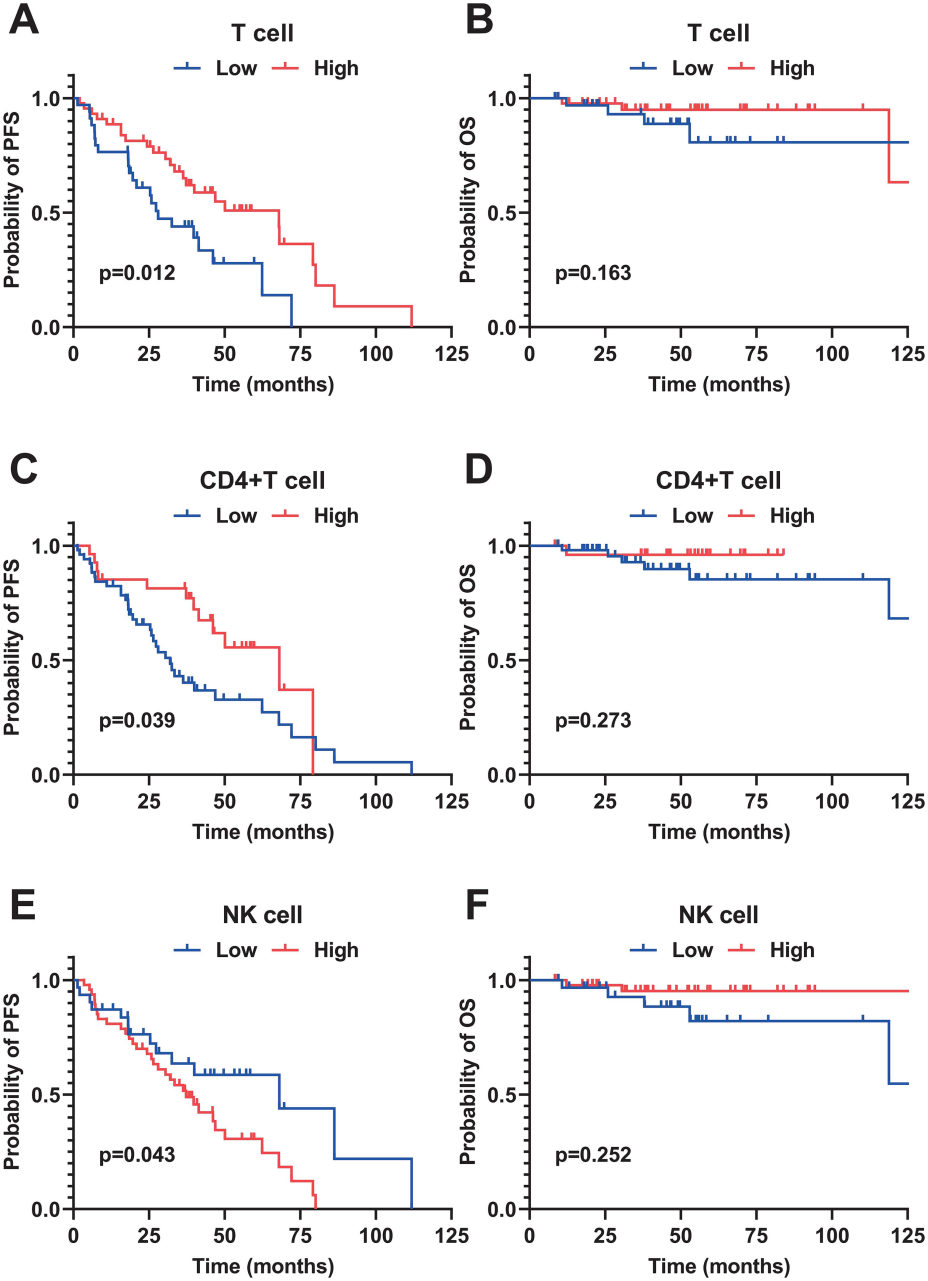
Kaplan–Meier survival curves of peripheral blood immune cell subsets in patients with MCL. **(A,B)** PFS and OS curves stratified by the percentage of total T cells (cutoff value = 70.42%). **(C,D)** PFS and OS curves stratified by the percentage of CD4^+^T cells (cutoff value = 34.63%). **(E,F)** PFS and OS curves stratified by the percentage of natural killer (NK) cells (cutoff value = 8.37%). All survival differences were analyzed by the Log-rank test. PFS, progression-free survival; OS, overall survival.

The median follow-up for patients in this cohort was 40 months. PFS rates at 1, 3, and 5 years were 36.0, 16.7, and 2.6%, respectively. OS rates for the same periods were 72.1, 53.8, and 17.9%. A univariate analysis of 78 patients with MCL identified several risk factors for poor PFS: elevated β2-MG (*p* = 0.05), Ann Arbor stage III-IV (*p* = 0.024), albumin levels below 35 g/L (*p* = 0.008), and a high SUVmax of ≥ 11 (*p* < 0.001). For OS, pleomorphic and blastoid pathological types (*p* = 0.002), low albumin levels (*p* = 0.018), and high SUVmax (*p* < 0.001) were significant risk factors. In examining immune characteristics, a low total T cell proportion (<70.42, *p* = 0.012), decreased CD4 + T cell proportion (<34.63, *p* = 0.039), and increased NK cell proportion (≥8.37, *p* = 0.043) were associated with poor PFS (Figure 1). A multivariate analysis focused on clinical characteristics with *p* < 0.1 revealed that a maximum standardized uptake value (SUVmax) of ≥ 11 on PET-CT was an independent risk factor associated with both poor PFS (HR = 1.475, 95% CI: 1.094–1.987, *p* = 0.011) and poor OS (HR = 1.74, 95% CI: 1.099–2.774, *p* = 0.018).

### 3.5 Establishment of a prognostic model based on immune function

**Figure 2:**
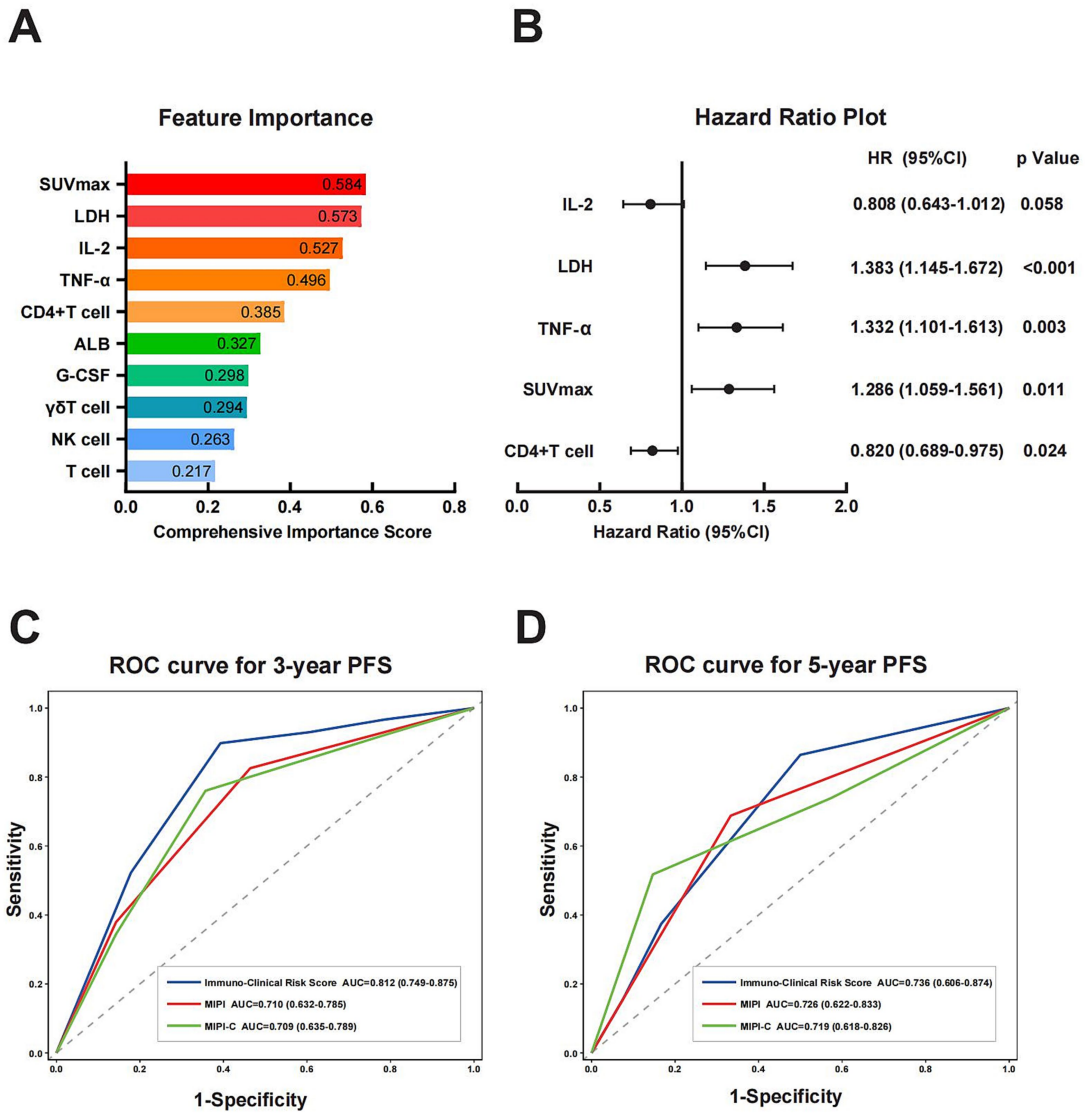
Construction of prognostic models and comparison of prognostic capabilities. **(A)** Ranking of feature importance based on separate XGBoost regression and classification models. The comprehensive importance score was calculated via weighted synthesis, with SUVmax, LDH, IL-2, TNF-α, and CD4^+^ T cells identified as the top five key features. **(B)** HR plot of key features derived from multivariate Cox regression analysis. **(C,D)** ROC curves analysis of Immuno-Clinical Risk Score, MIPI, and MIPI-C for 3-year and 5-year PFS prediction. The Immuno-Clinical Risk Score exhibited a higher AUC compared with MIPI and MIPI-C. SUVmax, maximum standardized uptake value; LDH, lactate dehydrogenase; IL-2, interleukin-2; TNF-α, tumor necrosis factor-α; HR, hazard ratio; ROC, receiver operating characteristic curve; AUC, area under the curve; MIPI, Mantle Cell Lymphoma International Prognostic Index.

We developed a prognostic model focused on immune function, using PFS as the endpoint. The model included 18 candidate features, comprising both immune function and clinical characteristics. Specifically, it incorporated 8 clinical features: Ki-67, pathological subtype, stage, B symptoms, ECOG score, albumin, LDH, and SUVmax from PET-CT at initial diagnosis. Additionally, it included 10 immune function features: T cells, CD4 + T cells, CD8 + T cells, γδT cells, NK cells, early and late activated T cells, TNF-*α*, IL-2, and G-CSF. The dataset was randomly divided into a training set and a test set in a 7:3 ratio. The training set was used for feature selection and model training, while the test set validated preliminary performance. All features were standardized using StandardScaler. We constructed separate XGBoost regression and classification models and assessed the contribution of each indicator through feature importance analysis. Then we calculated the comprehensive importance of features using a weighted synthesis method. The top 10 features’ importance is detailed in the Figure 2A. Among these, the five key features identified were SUVmax (0.584), LDH (0.573), IL-2 (0.527), TNF-*α* (0.496), and CD4 + T cell (0.385). These features were used to construct the prognosis model. A multivariate Cox regression analysis was performed on these five features (Figure 2B). Using the significance of the Cox regression coefficients, we established a formula to calculate each patient’s individual risk score. The risk score was determined by the linear summation of the product of the regression coefficient and the feature value. The specific formula is as follows:


$$\begin{array}{l} \text{Risk Score}=0.325\times \mathrm{LDH}/\mathrm{ULN}+0.252\times \text{SUVmax}\\ +0.287\times \mathrm{TNF}-\alpha (\mathrm{pg}/\mathrm{mL})-0.214\times \mathrm{IL}-2(\mathrm{pg}/\mathrm{mL})\\ -0.199\times \mathrm{CD}4+T\;\text{cell}(\%) \end{array}$$

**Figure 3:**
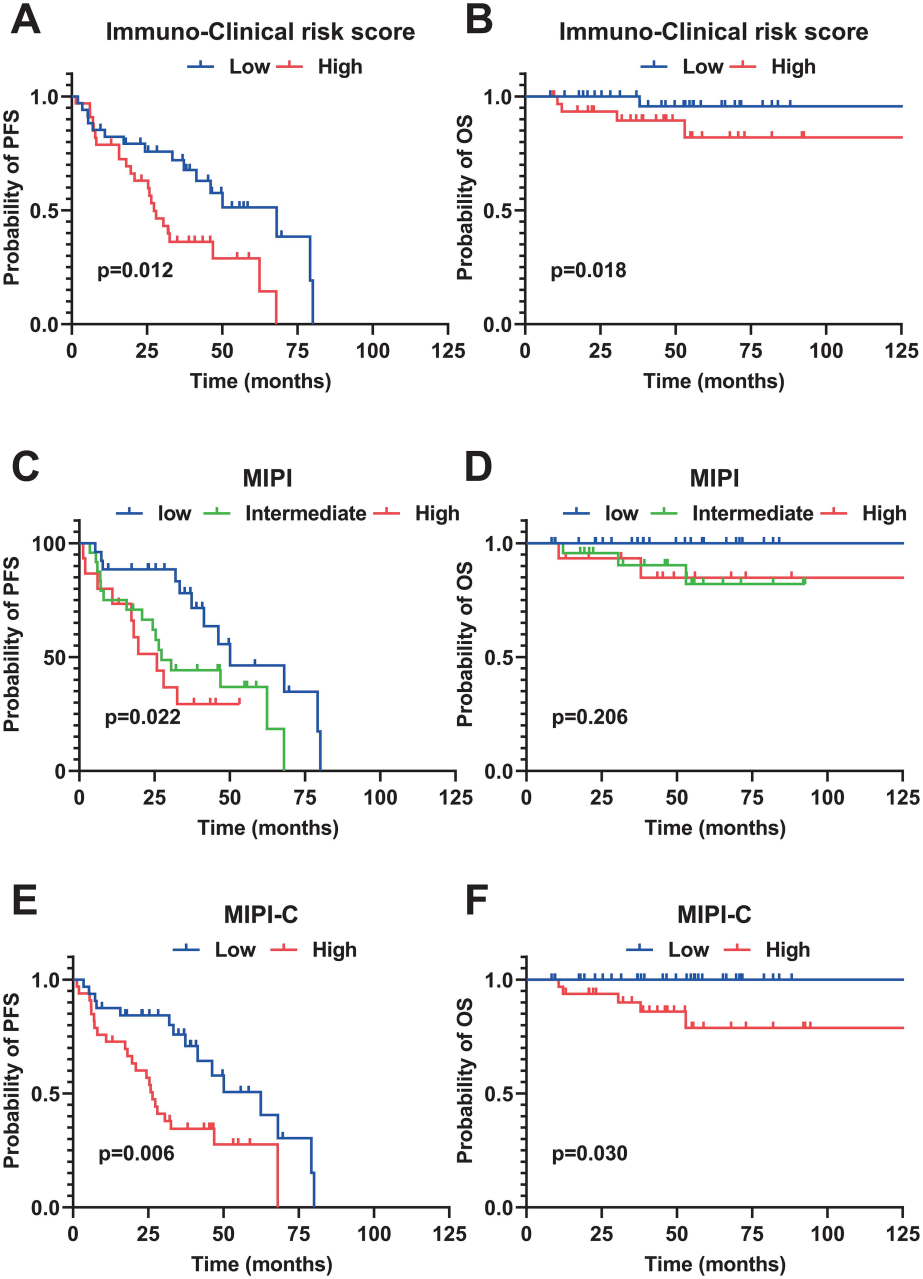
Kaplan–Meier survival curves of different prognostic model. **(A,B)** PFS and OS curves stratified by the Immuno-Clinical Risk Score (median cutoff value = 6.137). **(C,D)** PFS and OS curves stratified by MIPI. **(E,F)** PFS and OS curves stratified by MIPI-C. All survival analyses were performed using the Log-rank test. PFS, progression-free survival; OS, overall survival; MIPI, Mantle Cell Lymphoma International Prognostic Index.

Patients were divided into high-risk and low-risk groups using a median risk score of 6.137. Individuals scoring over 6.137 were deemed high-risk, whereas scores equal to or below 6.137 denoted low-risk. The concordance index (C-index) of the model was 0.680. Kaplan–Meier survival analysis showed significantly better PFS (*p* = 0.012) and better OS (*p* = 0.018) in the low-risk group compared to the high-risk group, indicating the model’s effectiveness in predicting PFS and OS (Figures 3A,B). Additionally, Kaplan–Meier analysis for PFS and OS was performed using the MIPI and MIPI-C models within this cohort. Both models demonstrated significant differences in PFS across their risk strata (Figures 3C–F). As for OS, distinct prognostic differences were observed among different risk strata of MIPI-C, whereas no significant prognostic differences were found in the risk stratification of MIPI. Overall, this immune-clinical model exhibited strong discriminatory power for PFS and OS compared to previous models.

**Figure 4:**
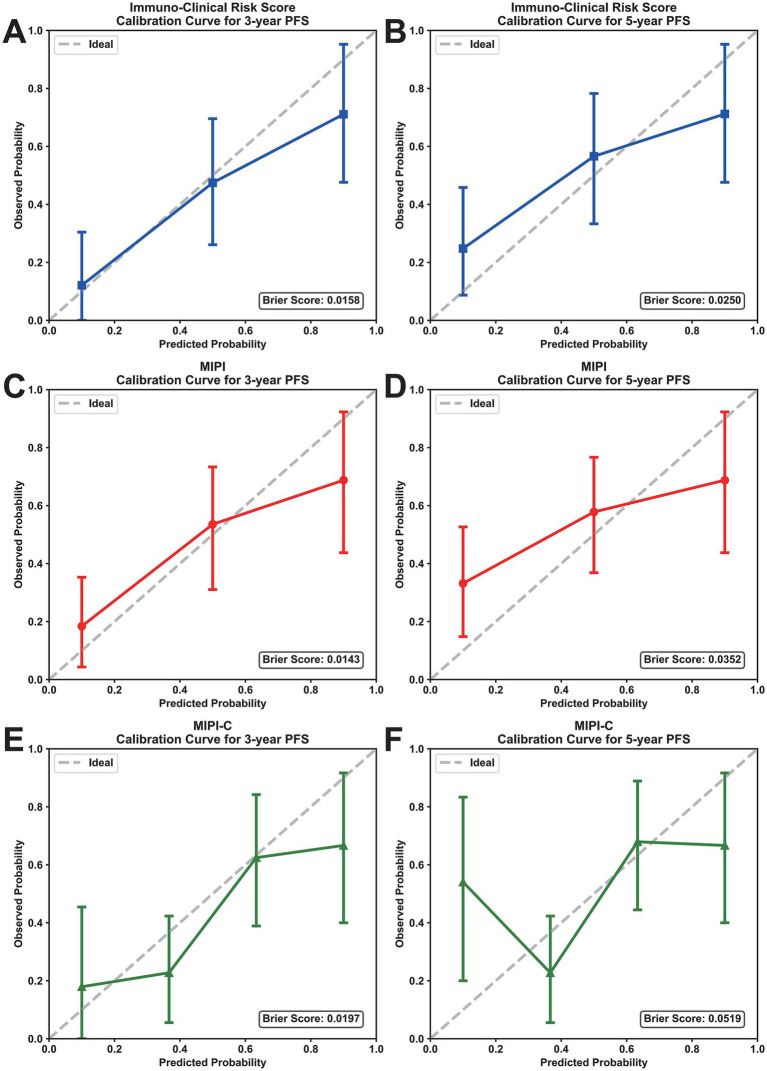
Calibration curves of prognostic models for PFS prediction. **(A,B)** Calibration curves for 3-year and 5-year PFS of the immuno-clinical risk score. **(C,D)** Calibration curves for 3-year and 5-year PFS of MIPI. **(E,F)** Calibration curves for 3-year and 5-year PFS of MIPI-C. The *x*-axis represents the predicted probability, and the *y*-axis represents the actual probability; the ideal line indicates perfect calibration. PFS, progression-free survival; MIPI, mantle cell lymphoma international prognostic index.

We evaluated the prognostic efficacy of our model using 1,000 iterations of repeated sampling verification with the Bootstrap method. The results showed that our model’s average area under the curve (AUC) for predicting 3-year PFS was 0.812 (95% CI: 0.749–0.875). In contrast, the MIPI score’s average AUC for predicting 3-year PFS was 0.710 (95% CI: 0.632–0.785), and the MIPI-C score’s average AUC was 0.709 (95% CI: 0.635–0.789). For predicting 5-year PFS, the average AUC of the new model was 0.736 (95% CI: 0.606–0.874), that of MIPI was 0.726 (95% CI: 0.622–0.833), and that of MIPI-C was 0.719 (95% CI: 0.618–0.826) (Figures 2C,D). These results suggest that our model provides superior prognostic value. To evaluate calibration, we plotted a calibration curve to compare predicted probabilities with actual outcomes (Figure 4). The curve for 3-year and 5-year PFS demonstrated that the consistency between predicted and actual values in our model was on par with the MIPI and MIPI-C scores. This underscores the reliability of our immune function-based prognostic model as a valuable tool.

## 4 Discussion

Mantle cell lymphoma (MCL) exhibits diverse clinical symptoms (1, 16). Traditional prognostic models mainly rely on standard clinical parameters, often neglecting the role of the immune microenvironment and the patient’s immune status (5, 17). As a result, these models do not fully account for how immune function affects prognosis. In our study, we retrospectively analyzed peripheral blood immune indicators and clinical data from 78 MCL patients. We thoroughly investigated the prognostic value of lymphocyte subsets and cytokines. Using machine learning techniques, we developed a prognostic model that incorporates immune features. This model not only confirms previous research findings but also quantifies clinical immune indicators, improving their utility in assessing MCL prognosis.

Research has increasingly focused on the link between immune cells and prognosis in MCL (Table 3). Nygren et al. analyzed lymph node samples from MCL patients, finding that higher levels of CD4 + T cells were associated with better clinical outcomes (13). This finding provides evidence supporting the prognostic significance of CD4 + T cells in MCL. Zhang et al. further expanded this research to peripheral blood, demonstrating that a low absolute CD4 + T cell count is associated with poor prognosis in patients with MCL. These findings support the clinical utility of peripheral blood immune markers for prognostic assessment in MCL (18). Zhou et al. conducted a retrospective analysis showing that a low absolute count of NK cells in peripheral blood correlated with reduced overall survival (OS) in MCL patients (19), suggesting that fewer NK cells may weaken the anti-tumor immune response. Li et al. used immunohistochemistry to demonstrate that a high density of CD68 + and CD163 + macrophages in the MCL tumor microenvironment is linked to poor OS (20), emphasizing the prognostic importance of local tumor immune cells. Zhu et al. found that CD3 + T cells are an biomarker for better prognosis (21). In the present study, stratified analysis by MIPI-C score revealed that 46.2% of patients in the low risk group had CD4 + T cell percentages >34.63%, whereas only 12.8% of patients in the high risk group exceeded this threshold (*p* = 0.021). This finding demonstrates an association between CD4 + T cells and traditional clinical risk stratification, confirming their role as a prognostic factor. Furthermore, the study identified a critical threshold of 34.63% for the CD4 + T cell proportion in peripheral blood as a prognostic marker for MCL. A CD4 + T cell proportion of ≤34.63% was significantly associated with poorer PFS (*p* = 0.039). The results are consistent with prior tumor microenvironment studies showing that higher CD4 + T cell level in peripheral blood and tumor microenvironment correlate with better prognosis in MCL (13).

**Table 3:** Previously reported immune cells for the prognosis of lymphoma.

Year	Lymphoma	Tissue source	Immune cells	Prognosis	References
2014	MCL	Lymph node	CD4 CD8 CD4/CD8	Better Better Better	(13)
2016	MCL	Peripheral blood	CD4 CD4/CD8	Better Better	(18)
2019	MCL	Peripheral blood	AMC NK	Worse Better	(19)
2021	MCL	Lymph node	M1 M2	Worse Worse	(20)
2022	MCL	Peripheral blood	CD4 CD8	Better Worse	(15)
2025	MCL	Tumor tissue	CD3	Better	(21)

B-NHL, B-cell Non-Hodgkin’s Lymphoma; MCL, Mantle Cell Lymphoma; CD4, CD4 + T cell; CD8, CD8 + T cell; CD4/CD8, Ratio of CD4 + T cell to CD8 + T cells; AMC, Absolute Monocyte Count; NK, Natural Killer Cell; M1, M1-type Macrophage; M2, M2-type Macrophage; CD3, CD3 + T cell.

Cytokines, as signaling molecules among immune cells, reflect the body’s immune status through their level variations. While most MCL related studies focused on immune cell components, research on cytokines’ prognostic significance in MCL is limited. This study explored the prognostic association of IL-2 and TNF-*α* in MCL. IL-2 promotes T cell and NK cell proliferation and activation, enhancing the anti-tumor immune response (22–24). In contrast, TNF-α, a pro-inflammatory cytokine, amplifies pro-inflammatory signals and anti-apoptotic regulators, supporting lymphoma tumor cell growth and proliferation (25). The study found that MCL patients with higher IL-2 levels were associated with better prognosis, whereas those with increased TNF-α levels were associated with worse prognosis. These findings align with the immunobiological roles of IL-2 and TNF-α.

However, although the peripheral blood findings in this study align with prior reports linking the tumor immune microenvironment to MCL prognosis, the relationship between peripheral immune parameters and the local tumor immune milieu warrants caution. Peripheral immune phenotypes do not fully reflect tissue-specific immune infiltration within tumors, and therefore cannot be treated as direct proxies for the tumor microenvironment. In addition, blood immune cell composition and cytokine levels are shaped by systemic factors such as infection and stress and are dynamically variable, which limits their reliability as stable prognostic biomarkers.

MIPI and MIPI-C are well-established prognostic models for MCL, with MIPI-C improving upon MIPI by incorporating Ki-67 to classify patients into four risk categories (6). However, both models fail to account for immune status, making it challenging to explain prognostic differences among patients with similar clinical characteristics. This study presented a novel prognostic model that integrates both clinical and immune features. Utilizing XGBoost, we identified five critical features: LDH, SUVmax, CD4 + T cells, IL-2, and TNF-*α*. Importantly, CD4 + T cells, IL-2, and TNF-α are immune-related markers. By incorporating immune markers with tumor functional imaging indicators like SUVmax, we significantly enhanced the model’s clinical relevance. After performing efficient feature selection using XGBoost, we assigned prognostic scores to the features through Cox regression analysis to enhance the interpretability of the prognostic model. Scoring was based on a simple linear formula derived from the Cox regression, effectively stratifying patients into distinct risk groups that exhibited significant differences in progression-free survival and overall survival outcomes. PET-CT and LDH are routine examinations for lymphoma patients, while peripheral blood immune function testing is more accessible, noninvasive, and repeatable compared with assessment of the tumor microenvironment. This risk stratification can be evaluated at initial diagnosis using readily available clinical indicators, making it applicable in clinical practice. Furthermore, this model showed superior discriminatory power compared to the MIPI and MIPI-C models and had comparable calibration ability, indicating its potential in predicting prognosis in MCL.

This study had several limitations. First, it was a single-center retrospective study with a small sample size and lacks external validation. To verify the model’s effectiveness, a multi-center prospective cohort study is required. Second, the study exclusively examined peripheral blood immune indicators, and the relationship between peripheral blood immune function and the tumor immune microenvironment is not definite. Future research should include immune cell analysis from tumor tissues to investigate the relationship between the tumor immune microenvironment and peripheral blood immune function. This approach could reveal the specific mechanisms by which immune cells and factors affect the progression of MCL, providing mechanistic insights for targeted MCL therapy.

This work supported existing prognosis studies on MCL and showed that CD4 + T cell, NK cell, IL-2, TNF-*α* were good prognostic markers. It also found important thresholds for CD4 + T cells (34.63%), IL-2 (1.89 pg./mL) and TNF-α (2.16 pg./mL). The immune-clinical integration model developed in this work is discriminatory and has preliminary predictive potential for the prognosis of MCL. This model and the identified immune associations warrant validation in future multicenter prospective studies and may inform further research into the role of immunity in MCL progression.

## Data Availability

The raw data supporting the conclusions of this article will be made available by the authors, without undue reservation.

## References

[1] Armitage JO Longo DL. Mantle-cell lymphoma. N Engl J Med. (2022) 386:2495–506. doi: 10.1056/NEJMra2202672, 35767440

[2] Cortelazzo S Ponzoni M Ferreri AJM Dreyling M. Mantle cell lymphoma. Crit Rev Oncol Hematol. (2020) 153:103038. doi: 10.1016/j.critrevonc.2020.103038, 32739830

[3] Eyre TA Bishton MJ McCulloch R O’Reilly M Sanderson R Menon G . Diagnosis and management of mantle cell lymphoma: a British Society for Haematology guideline. Br J Haematol. (2024) 204:108–26. doi: 10.1111/bjh.19131 37880821

[4] Hill HA Qi X Jain P Nomie K Wang Y Zhou S . Genetic mutations and features of mantle cell lymphoma: a systematic review and meta-analysis. Blood Adv. (2020) 4:2927–38. doi: 10.1182/bloodadvances.2019001350, 32598477 PMC7362354

[5] Hoster E Dreyling M Klapper W Gisselbrecht C van Hoof A Kluin-Nelemans HC . A new prognostic index (MIPI) for patients with advanced-stage mantle cell lymphoma. Blood. (2008) 111:558–65. doi: 10.1182/blood-2007-06-095331, 17962512

[6] Hoster E Rosenwald A Berger F Bernd H-W Hartmann S Loddenkemper C . Prognostic value of Ki-67 index, cytology, and growth pattern in mantle-cell lymphoma: results from randomized trials of the European mantle cell lymphoma network. J Clin Oncol. (2016) 34:1386–94. doi: 10.1200/JCO.2015.63.8387, 26926679

[7] Balsas P Veloza L Clot G Sureda-Gómez M Rodríguez M-L Masaoutis C . SOX11, CD70, and Treg cells configure the tumor immune microenvironment of aggressive mantle cell lymphoma. Blood. (2021) 138:2202–15. doi: 10.1182/blood.2020010527, 34189576 PMC8641098

[8] Saleh K Cheminant M Chiron D Burroni B Ribrag V Sarkozy C. Tumor microenvironment and immunotherapy-based approaches in mantle cell lymphoma. Cancer. (2022) 14:3229. doi: 10.3390/cancers14133229, 35804999 PMC9265015

[9] Zelenetz AD Gordon LI Abramson JS Advani RH Andreadis B Bartlett NL . NCCN guidelines® insights: B-cell lymphomas, version 6.2023. J Natl Compr Cancer Netw. (2023) 21:1118–31. doi: 10.6004/jnccn.2023.0057, 37935098

[10] Brudno JN Maus MV Hinrichs CS. CAR T cells and T-cell therapies for Cancer: a translational science review. JAMA. (2024) 332:1924–35. doi: 10.1001/jama.2024.19462, 39495525 PMC11808657

[11] Wallace D Reagan PM. Novel treatments for mantle cell lymphoma: from targeted therapies to CAR T cells. Drugs. (2021) 81:669–84. doi: 10.1007/s40265-021-01497-y, 33783717

[12] Qualls D Kumar A Epstein-Peterson ZD. Targeting the immune microenvironment in mantle cell lymphoma: implications for current and emerging therapies. Leuk Lymphoma. (2022) 63:2515–27. doi: 10.1080/10428194.2022.2086244, 35704674 PMC9741766

[13] Nygren L Wasik AM Baumgartner-Wennerholm S Jeppsson-Ahlberg Å Klimkowska M Andersson P . T-cell levels are prognostic in mantle cell lymphoma. Clin Cancer Res. (2014) 20:6096–104. doi: 10.1158/1078-0432.CCR-14-0889 25294911

[14] Gergely L Váncsa A Miltényi Z Simon Z Baráth S Illés Á. Pretreatment T lymphocyte numbers are contributing to the prognostic significance of absolute lymphocyte numbers in B-cell non-Hodgkins lymphomas. Pathol Oncol Res. (2011) 17:249–55. doi: 10.1007/s12253-010-9306-2, 20842470

[15] Lv H Fei Y Li W Wang Y Wang J He J . A novel clinical immune-related prognostic model predicts the overall survival of mantle cell lymphoma. Hematol Oncol. (2022) 40:343–55. doi: 10.1002/hon.2994, 35368100

[16] Dreyling M Campo E Hermine O Jerkeman M Le Gouill S Rule S . Newly diagnosed and relapsed mantle cell lymphoma: ESMO clinical practice guidelines for diagnosis, treatment and follow-up. Ann Oncol. (2017) 28:iv62–71. doi: 10.1093/annonc/mdx223, 28881919

[17] Hill HA Jain P Ok CY Sasaki K Chen H Wang ML . Integrative prognostic machine learning models in mantle cell lymphoma. Cancer Res Commun. (2023) 3:1435–46. doi: 10.1158/2767-9764.CRC-23-0083, 37538987 PMC10395375

[18] Zhang X-Y Xu J Zhu H-Y Wang Y Wang L Fan L . Negative prognostic impact of low absolute CD4+ T cell counts in peripheral blood in mantle cell lymphoma. Cancer Sci. (2016) 107:1471–6. doi: 10.1111/cas.13020, 27465799 PMC5084668

[19] Zhou X-H Zhang X-Y Liang J-H Zhu H-Y Wang L Xia Y . Low absolute NK cell counts in peripheral blood are associated with inferior survival in patients with mantle cell lymphoma. Cancer Biomark. (2019) 24:439–47. doi: 10.3233/CBM-182193, 30932881 PMC13082532

[20] Li P Yuan J Ahmed FS McHenry A Fu K Yu G . High counts of CD68+ and CD163+ macrophages in mantle cell lymphoma are associated with inferior prognosis. Front Oncol. (2021) 11:701492. doi: 10.3389/fonc.2021.701492, 34527580 PMC8435777

[21] Zhu N Wang Y Hu J Lin X Zhang C Tang F . Development of a novel prognostic model for mantle cell lymphoma based on quantitative detection of CD3 by quantitative dot blot. Front Oncol. (2025) 15:1595572. doi: 10.3389/fonc.2025.1595572, 41001043 PMC12457178

[22] Marr B Jo D Jang M Lee S-H. Cytokines in focus: IL-2 and IL-15 in NK adoptive cell Cancer immunotherapy. Immune Netw. (2025) 25:e17. doi: 10.4110/in.2025.25.e17, 40342841 PMC12056295

[23] Yang Z-Z Grote DM Ziesmer SC Manske MK Witzig TE Novak AJ . Soluble IL-2Rα facilitates IL-2–mediated immune responses and predicts reduced survival in follicular B-cell non-Hodgkin lymphoma. Blood. (2011) 118:2809–20. doi: 10.1182/blood-2011-03-340885, 21719603 PMC3172797

[24] Jiang T Zhou C Ren S. Role of IL-2 in cancer immunotherapy. Onco Targets Ther. (2016) 5:e1163462. doi: 10.1080/2162402X.2016.1163462, 27471638 PMC4938354

[25] D’mello KP Zhao L Kaser EC Zhu Z Xiao H Wakefield MR . The role of interleukins and the widely studied TNF-α in non-Hodgkin’s lymphoma. Med Oncol. (2021) 38:56. doi: 10.1007/s12032-021-01504-y, 33835307

