# Correction: A novel immune model predicts the prognosis of mantle cell lymphoma

**DOI:** 10.3389/fmed.2026.1844135

**Published:** 2026-04-16

**Authors:** Xiaoyu Hao, Yuqi Zhang, Wenxin Qi, Mingxia Zhu, Jing Wang, Ping Yang, Weilong Zhang, Hongmei Jing

**Affiliations:** Department of Hematology, Peking University Third Hospital, Beijing, China

**Keywords:** biomarker, immune function, machine learning, mantle cell lymphoma, prognosis

The prognostic risk score calculation formula at the end of **Results**, *3.5 Establishment of a prognostic model based on immune function*, Paragraph 1 was erroneously given as:

“Risk Score = 0.325 × LDH(IU / L) + 0.252 × SUVmax +0.287 × TNF-α(pg / mL)-0.214 × IL-2(pg / mL)−0.199 × CD 4+T cell(%)”

The correct formula is:

“Risk Score = 0.325 × LDH/ULN + 0.252 × SUVmax +0.287 × TNF-α(pg/mL) – 0.214 × IL-2(pg/mL) – 0.199 × CD4+ T cell(%)”

The original version of this article has been updated.

